# Associations of the Colon Tissue Microbiome and Circulating Bile Acids With Colorectal Adenoma Among Average‐Risk Women

**DOI:** 10.1002/cam4.71048

**Published:** 2025-09-12

**Authors:** Doratha A. Byrd, Maria F. Gomez, Stephanie R. Hogue, Jessica R. Burns, Nate Smith, Joshua Sampson, Erikka Loftfield, Patricia G. Wolf, Yunhu Wan, Andrew Warner, Belynda Hicks, Casey Dagnall, Kristine Jones, Youngchul Kim, Jin Xu, Jianxin Shi, Rashmi Sinha, Emily Vogtmann

**Affiliations:** ^1^ Department of Cancer Epidemiology H. Lee Moffitt Cancer Center and Research Institute Tampa Florida USA; ^2^ Department of Gastrointestinal Oncology H. Lee Moffitt Cancer Center and Research Institute Tampa Florida USA; ^3^ College of Public Health, University of South Florida Tampa Florida USA; ^4^ Non‐Therapeutic Research Office H. Lee Moffitt Cancer Center and Research Institute Tampa Florida USA; ^5^ Division of Cancer Epidemiology and Genetics National Cancer Institute, National Institute of Health Rockville Maryland USA; ^6^ Department of Nutrition Science Purdue University West Lafayette Indiana USA; ^7^ Leidos Biomedical Research, Inc., Frederick National Laboratory for Cancer Research Frederick Maryland USA; ^8^ Department of Biostatistics and Bioinformatics H. Lee Moffitt Cancer Center & Research Institute Tampa Florida USA

**Keywords:** adenoma, alpha diversity, microbiome

## Abstract

**Objective:**

The gut microbiome and bile acids (BAs) likely influence colorectal cancer (CRC) development and disparities. We conducted a nested case–control study of the associations of the colon tissue microbiome and circulating BAs with colorectal adenoma prevalence in the previously conducted multi‐center Colorectal Neoplasia Screening with Colonoscopy in Average‐Risk Women Regional Navy/Army Medical Centers study (CONCeRN).

**Methods:**

We individually matched 143 women with adenoma to 279 without adenoma. Using 16S rRNA gene sequencing, we assessed alpha and beta diversity, taxonomic abundance, and co‐abundance groups (CAGs). Fasting serum was analyzed for 13 primary and secondary BAs.

**Results:**

The presence of oral‐originating *Porphyromonas* was positively associated with adenomas (odds ratio [OR] and 95% confidence interval [CI] = 2.50 [1.18, 5.30]; *p =* 0.02). Race and study center explained statistically significant percentages of variation in the beta diversity matrices. BAs were generally positively associated with adenomas, though these results were not statistically significant.

**Discussion:**

Overall, our findings suggest the colon tissue microbiome may differ by race and geography, and that certain oral‐originating bacteria may be positively associated with adenomas.

## 1 Introduction

Colorectal cancer (CRC), the second leading cause of cancer death in the United States [1], poses a disproportionate burden among Black women, who have an 18% higher rate of CRC incidence and a 31% higher risk of CRC mortality compared to White women [2]. The trillions of microbes in the gut have many plausible roles in CRC etiology and disparities. Numerous epidemiologic and basic science studies have investigated a role of the gut microbiome in CRC [3, 4], including multiple case–control studies of the tissue microbiome [5] and colorectal neoplasms [6]. In comparison with its luminal fecal counterparts, the mucosa may reflect the colonic bacterial community that inhabits the epithelial crypts [7]. In a meta‐analysis of four case–control studies comparing the microbiome in 16S ribosomal RNA (rRNA) gene sequenced tumor tissue or normal tissue collected, respectively, from CRC cases and healthy controls, the relative abundance of *Bacteroides fragilis* , *Fusobacterium nucleatum* , *Parvimonas micra* , and *Peptostreptococcus stomatis* —the latter three being known oral pathogens—was higher within the CRC tumor samples [5].

The associations of the gut microbiome with CRC risk may be mediated in part by microbiome‐related metabolites. The liver produces primary bile acids (BAs), which can be conjugated to glycine or taurine to increase water solubility prior to being excreted into the biliary tract. Conjugated BAs can then be deconjugated by gut microbes via the bacterial enzyme bile salt hydrolase and transformed into secondary BAs via 7‐alpha‐dehydroxylation [8]. BAs exhibit potential carcinogenic properties; we previously found that circulating BAs were associated with a higher risk of adenoma recurrence [3, 9]. Overall, there is accumulating evidence for interactions of the gut microbiome with BAs, but there is a need to better characterize the associations of the microbiome and BAs with CRC precursors and the potential role of these exposures in excess CRC burden.

Taken together, there is accumulating evidence for the role of the gut microbiome and its metabolites in CRC. Therefore, we investigated associations of normal colon tissue microbiome and circulating BAs with prevalent colorectal adenomas in a case–control study nested within The Colonoscopy in Average‐Risk Women Regional Navy/Army Medical Centers study (CONCeRN).

## 2 Methods

### 2.1 Study Population

CONCeRN, described in detail previously [10, 11], was a multicenter screening study designed to evaluate the efficacy of colonoscopy versus sigmoidoscopy for CRC screening among asymptomatic, average‐risk women. Women were referred for CRC screening at four tertiary‐care, military medical centers (National Naval Medical Center, Bethesda, MD; Walter Reed Army Medical Center, Washington, DC; and Naval Medical Centers in San Diego, CA and Portsmouth, VA) and recruited from July 1999 through December 2002. Typically, these centers serve those in the military, their families, and military retirees. Eight hundred thirteen women, aged 50–79 years or 40–79 years with a family history of CRC among a first‐degree relative, participated in an etiologic component of the study involving additional biospecimen and questionnaire collection. To ensure recruitment of asymptomatic women at average risk, exclusion criteria included normal findings on colonoscopy or barium enema within the preceding 10 years or sigmoidoscopy within the preceding 5 years, positive fecal occult blood tests (FOBT) or iron‐deficiency anemia within the preceding 6 months, unintentional weight loss of more than 10 pounds in recent years, hereditary nonpolyposis colorectal cancer syndrome, familial adenomatous polyposis, or a history of adenoma, CRC, or inflammatory bowel disease. The study was approved by institutional review boards at the NCI and participating medical centers. All participants provided written informed consent.

### 2.2 Case Ascertainment

After standard bowel preparation, all subjects in the colonoscopy arm of the study underwent a complete colonoscopy, which reached the cecum in 98.7% of the women. For women with observed polyps, the location was defined based on the depth of insertion of the colonoscope and anatomic landmarks (hepatic flexure, splenic flexure, and junction of the descending and sigmoid colon), and the diameter was estimated by a guide wire (Olympus Colonoscopy Measuring Guidewire; Olympus America, Center Valley, PA). Final diagnosis of each polyp was made by an expert gastrointestinal pathologist who reviewed histologic specimens without knowledge of the initial diagnosis during colonoscopy. Based on the most advanced lesion found in the entire colon and rectum, cases of adenoma were defined as those with a pathologically verified adenomatous polyp of any histological type, size, or location in the colon or rectum, excluding hyperplastic polyps or benign lesions. Advanced adenoma cases were defined as those having high‐grade dysplasia, any adenoma with a villous component, or large adenomas (≥ 1 cm) [12].

### 2.3 Data Collection

Participants completed a self‐administered 124‐item validated Diet History Questionnaire (DHQ) and a risk factor questionnaire ascertaining demographic and lifestyle characteristics, including self‐reported race and ethnicity, family cancer history, and anthropometrics.

### 2.4 Microbiome Assessment of Colonic Biopsies

During colonoscopy, the endoscopist removed six normal colonic mucosa biopsies in the region of the splenic flexure from each woman enrolled in the etiological portion of the study. The study nurse in the endoscopy suite immediately snap froze the biopsies using liquid nitrogen, placed the frozen specimens on dry ice, and transferred them to a biorepository facility where they were stored at −70°C. A subset of these biopsies was previously used to assess colonic folate concentration [10]. We selected *N* = 176 adenoma cases with ≥ 1 biopsy sample for the microbiome study. We selected *N* = 352 individuals without an adenomatous polyp to serve as controls for the microbiome analyses. Two controls were matched to each case on 5‐year age group and month and study site of colonoscopy. A total of 94 study samples were removed from the analysis as described below. A second colonic biopsy from 50 cases and 50 controls was included to assess technical reproducibility.

Matched cases and controls, and their replicates were included in the same batch. In addition, three quality control samples were included in each batch: [13] 1) an artificial community sample; 2) a robogut sample; and 3) an extraction blank. The biopsy tissues were lysed using an enzymatic cocktail, homogenized in a Bead Ruptor (Omni International Inc., Kennesaw, GA, USA), and centrifuged. The Animal Tissue DNA Extraction Kit (AutoGen, Holliston, MA, USA) was used for DNA extraction following the standard protocol. After DNA extraction, 888 of 898 study samples and quality control samples qualified for 16S rRNA gene sequencing based on DNA concentration. In the blank extraction samples, there was a median of 2505 reads across 20 blank samples (one blank remained after rarefaction). The artificial community and robogut samples tended to cluster in PCoA plots (Figure S1). The coefficients of variation for observed amplicon sequence variants (ASVs), the Shannon Index, and Faith's phylogenetic diversity (PD) ranged 1.48%–17.03% for the artificial communities and 1.81%–9.58% for robogut samples, suggesting relatively low variability between DNA extraction batches (Table S1).

The 16S rRNA gene PCR amplification and sequencing was performed as previously described [14]. Twenty nanogram, at a concentration of 1 ng/μL, was used as input for PCR amplification, based on Quant‐iT PicoGreen double‐stranded (ds) DNA (Thermo Fisher Scientific, Grand Island, NY) quantitation. The V4 region of the 16S rRNA gene was PCR amplified, with cycle number increased to 35 cycles to account for low microbial content in tissue‐derived DNA. 2 × 250 bp paired end sequencing was performed on the Illumina MiSeq using the 500 cycle v2 kit (Illumina, San Diego, CA, USA).

Using the Divisive Amplicon Denoising Algorithm (DADA) 2 pipeline 1.2.1 [15], sequence variant tables and phylogenetic trees were generated based on pair‐end sequence reads. For quality filtering, the first 10 bases were trimmed from forward and reverse reads. Forward/reverse reads were truncated at 240/220 bases separately. Then, the reads were merged using the default “mergePairs” DADA2 function. After merging and error correction, amplicon sequence variants (ASVs; i.e., 100% operational taxonomic units, or OTUs) were identified. Taxonomy was assigned to the resulting ASVs using the SILVA v123 database [16].

We estimated standard alpha diversity (e.g., observed ASVs, Shannon Index, and Faith's PD) and beta diversity (e.g., weighted and unweighted UniFrac and Bray–Curtis) metrics. We determined the rarefaction threshold for alpha and beta diversity estimates empirically based on a balanced assessment of rarefaction curves and retention of study samples with a final rarefaction level of 11,000 reads (Figure S2), and confirmed similar results at a lower rarefaction of 5000 reads. For any controls removed, all corresponding matched adenoma cases were kept if the case had at least one matching control remaining. If a case was removed, the matched controls were also removed. At this rarefaction level, there were 94 study samples removed from the analysis (Table S2). All alpha diversity estimates were calculated based on the average of 10 rarefaction samplings. The first principal coordinate axes explained 12.34%, 8.59%, and 15.15% of variation in the beta diversity matrix for Bray–Curtis, unweighted UniFrac, and weighted UniFrac, respectively. We assessed the relative abundance and presence/absence of bacterial taxa, with a focus on a priori‐selected bacteria hypothesized to be associated with CRC (e.g., *Bacteroides*, *Fusobacterium*, *Porphyromonas*, *Parvimonas*, *Peptostreptococcus*, *Gemella*, *Prevotella*, *Solobacterium*, *Dialister*, and *Clostridiales* [Order]) as demonstrated by a meta‐analysis of the tissue microbiome and CRC [5, 6]. For these analyses and the exploratory analyses of relative abundance, we focused on bacteria that were present in ≥ 50% of samples at an average relative abundance of ≥ 0.01%. For exploratory analyses of the presence of bacteria, we focused on bacteria that were present in 5%–95% of the population. To account for the compositional nature of the microbial abundance, we transformed the relative abundance with the centered log ratio transformation in our primary analyses [17] and compared these associations with relative abundances multiplied by 100 (both were standardized to a mean of 0 and standard deviation of 1). Finally, we inferred microbial guilds that can better represent overall community functionality and be more relevant than a single bacteria‐adenoma model, as described previously [18]. A total of 363 bacterial ASVs present in at least 5% of the samples were clustered into co‐abundance groups (CAGs) by using a hierarchical cluster analysis based on SparCC co‐abundance correlations as a taxon‐wise distance metric [19]. The optimal number of CAGs was determined by examining the gap statistic [20] and resulted in 17 guilds.

In participants with microbiome data from more than one colon tissue biopsy, we compared the colon tissue microbiome of each biopsy to assess the reliability of samples collected consecutively from the splenic flexure to inform analyses and interpretation of bacterial profiles in single versus multiple biopsy specimens. We calculated intraclass correlation coefficients (ICCs) for alpha diversity, beta diversity, and dominant phyla/genera using linear mixed effects models with a random effect for the participant. Overall, we found high concordance (Table S3) with ICCs generally > 75% except for *Prevotella*, so we randomly selected one replicate to include for the microbiome analysis described below.

### 2.5 Bile Acid Assessment

We selected *N* = 164 cases with fasted serum collected prior to colonoscopy for the BA analysis and *N* = 328 controls matched one case per two controls on 5‐year age group and month and study site of colonoscopy (*N* = 154 cases and *N* = 293 controls also had microbiome data). For the BA measurements, matched cases and controls were included in the same batch. For quality control, we included three replicates from two different sources of pooled serum in each of the eight batches. BA concentrations in serum samples were analyzed by Metabolon using targeted assays for 13 of the most abundant primary and secondary BAs and their respective glycine and taurine conjugates. In brief, serum samples were spiked with a solution of corresponding labeled internal standards for each of the BAs and were subjected to protein precipitation with acidified methanol. Samples were then centrifuged, and a portion of the clear supernatant evaporated to dryness in a gentle stream of nitrogen at 40°C. The dried extract was then reconstituted, and an aliquot injected onto an Agilent 1290/Sciex QTrap 6500 mass spectrometer liquid chromatography tandem mass spectrometry (LC–MS/MS) system equipped with a C18 reverse phase HPLC column with acquisition in negative ion mode. The peak area of each parent or product ion was then measured against the peak area of the respective internal standard parent or product ion. Quantitation was then performed using least squares regression analysis generated from fortified calibration standards prepared immediately prior to each run.

Values for BA concentrations below the limit of detection were replaced by the lowest detected value for that metabolite divided by 2 [21]. Across the individual BAs, a median of 12% of samples were below the limit of detection (range 0.2%–21%). We created summary BA scores for broader BA groups, including total, primary, and secondary BAs. To do this, we summed the individual BA values and transformed the BA score by the natural logarithm (ln). Coefficients of variation based on the two sources of pooled serum included in each batch were below 15%, except for taurolithocholic and tauroursodeoxycholic, which were excluded from analysis. CVs for the other BAs ranged 1.56%–11.71% (Table S4).

### 2.6 Statistical Analysis

We summarized and compared participant characteristics by adenoma case/control status using chi‐square tests for categorical variables, analysis of variance (ANOVA) for continuous variables, and Kruskal–Wallis tests to perform nonparametric ANOVA for non‐normally distributed continuous variables.

We performed multivariate analyses to estimate the percentage of variation (*R* [2]) explained in the beta diversity matrices by race and other factors potentially associated with the microbiome (e.g., age, study site, diet [assessed via the Healthy Eating Index] [22], physical activity, body mass index, and others) using *adonis* from the R package vegan. We used multivariable conditional logistic regression to estimate odds ratios (ORs) and 95% confidence intervals (CIs) for the associations of the colon tissue microbiome metrics (alpha and beta diversity, relative abundance at the genus‐level and of CAGs, and genus‐level prevalence) and circulating BA concentrations with adenoma. We divided alpha diversity and BAs into tertiles based on the distribution within the controls. We calculated the *p*‐trend by assigning the median value of the tertile to the individual and entering the term continuously into the regression models. To test for overall differences in microbiome composition by adenoma presence, we conducted the microbiome regression‐based kernel association test (MiRKAT) [23], based on 10,000 permutations, to calculate *p* values based on kernel similarity matrices for Bray–Curtis and unweighted and weighted UniFrac distance, individually and overall. To test for heterogeneity by adenoma characteristics, we conducted a case‐only multivariable logistic regression analysis with adenoma characteristic as the dependent variable, and took the *p* value for the continuous alpha diversity metric to be the *P*_heterogeneity_. We estimated correlations of BAs with the microbiome metrics using partial Pearson correlations. Consideration for inclusion of covariates in the above‐described multivariable models was prespecified based on biological plausibility and previous literature and included known CRC‐ or microbiome‐related factors like age, year of colonoscopy, regular nonsteroidal anti‐inflammatory drug and aspirin use, alcohol use, family history of polyps, education, hormone replacement therapy, daily fiber intake, body mass index, total daily energy intake, physical activity level, race, smoking status, red and processed meat intake, and study center.

All statistical analyses were conducted in *R* version 4.3.1. We used Bonferroni correction to account for multiple testing by the number of statistical comparisons (i.e., alpha level = 0.05/N comparisons) for all exploratory analyses and used an alpha level of *p* = 0.05 for the a priori analysis.

## 3 Results

Within the 165 adenoma cases and 311 matched controls with microbiome data, cases were less likely to use aspirin and hormone replacement therapy, less likely to be White, and had, on average, a higher body mass index (BMI) compared with controls (Table 1).

**TABLE 1:** Characteristics of cases and controls in the Colorectal Neoplasia Screening with Colonoscopy in Average‐Risk Women Regional Navy/Army Medical Centers microbiome study, 2000–2002.

		Cases, *N* = 165			Controls, *N* = 311	
	*N* ^a^	(%)	Mean (SD)	*N*	(%)	Mean (SD)
Age			60.91 (9.02)			59.44 (8.59)
Race						
White	118	71.5		248	79.7	
Black	25	15.2		31	10	
Other	22	13.3		32	10.3	
Ethnicity						
Non‐Hispanic	161	97.6		305	98.1	
Hispanic	4	2.4		6	1.9	
BMI			27.74 (5.74)			26.55 (5.32)
Family history of polyps						
No	123	74.5		240	77.2	
Yes	42	25.5		71	22.8	
Aspirin/NSAID use						
No	101	61.2		151	48.6	
Yes	64	38.8		160	51.4	
HRT						
No	80	48.5		112	36.0	
Yes	85	51.5		199	64.0	
Smoking status						
Nonsmoker/missing	90	54.5		189	60.8	
Former/current smoker	75	45.5		122	39.2	
Alcohol use						
Nondrinker	98	59.4		172	55.3	
Drinker	67	40.6		139	44.7	
Center						
Bethesda	106	64.2		216	69.5	
Portsmouth	14	8.5		22	7.1	
San Diego	24	14.5		49	15.8	
Walter Reed	21	12.7		24	7.7	
Education						
≤ high school	45	27.3		66	21.2	
≥ some college	120	72.7		245	78.8	
Physical activity level						
None/unknown	30	18.2		62	19.9	
Moderate	79	47.9		142	45.7	
Vigorous	56	33.9		107	34.4	
Fiber intake (g/day)			11.18 (3.77)			11.50 (3.77)
Red meat and processed food intake (g/day)			42.54 (33.65)			39.46 (30.15)
Total energy intake (kcal/day)			1491 (628)			1538 (676)
Adenoma type^b^						
Normal	98	59.4				
Advanced	67	40.6				
Adenoma location^c^						
Distal	68	41.2				
Proximal	97	58.8				
Adenoma size						
Small adenoma (< 1 cm)	131	79.4				
Large adenoma (≥ 1 cm)	34	20.6				
A priori bacteria relative (%) abundance						
*Bacteroides*			26.31 (10.75)			26.05 (11.37)
*Fusobacterium*			1.65 (6.17)			1.28 (5.77)
*Gemella*			0.11 (0.28)			0.12 (0.39)
*Clostridiales* (Order)			57.37 (12.80)			56.85 (11.66)
Bile acid concentrations^d^						
Total			7.61 (1.68)			7.51 (1.68)
Primary			7.06 (1.74)			6.96 (1.69)
Secondary			5.22 (1.91)			4.92 (2.23)

Abbreviations: BMI, body mass index; CRC, colorectal cancer; HRT, hormone replacement therapy; NSAID, nonsteroidal anti‐inflammatory drug.

^a^ 154 cases and 293 controls have bile acid data.

^b^ Advanced adenomas are defined as having ≥ 1 adenoma with: a villous component, ≥ 10 mm diameter, high grade dysplasia, and/or having multiple adenomas.

^c^ Distal adenomas were defined as the most advanced adenoma being in the splenic flexure, descending, or sigmoid colon or rectum; proximal adenomas were defined as the most advanced adenoma being located in the cecum, ascending, hepatic flexure, or transverse colon.

^d^ Primary Bile Acids = log_2_ of the sum of chenodeoxycholic acid, cholic acid, glycocholic acid, glycochenodeoxycholic acid, taurocholic acid, and taurochenodeoxycholic acid; secondary Bile Acids = log_2_ of the sum of deoxycholic acid, lithocholic acid, glycodeoxycholic acid, glycolithocholic acid, and taurodeoxycholic acid. There are 154 cases and 293 controls with microbiome and bile acid data.

### 3.1 Colon Tissue Microbiome and Circulating BA Associations With Adenoma

**FIGURE 1:**
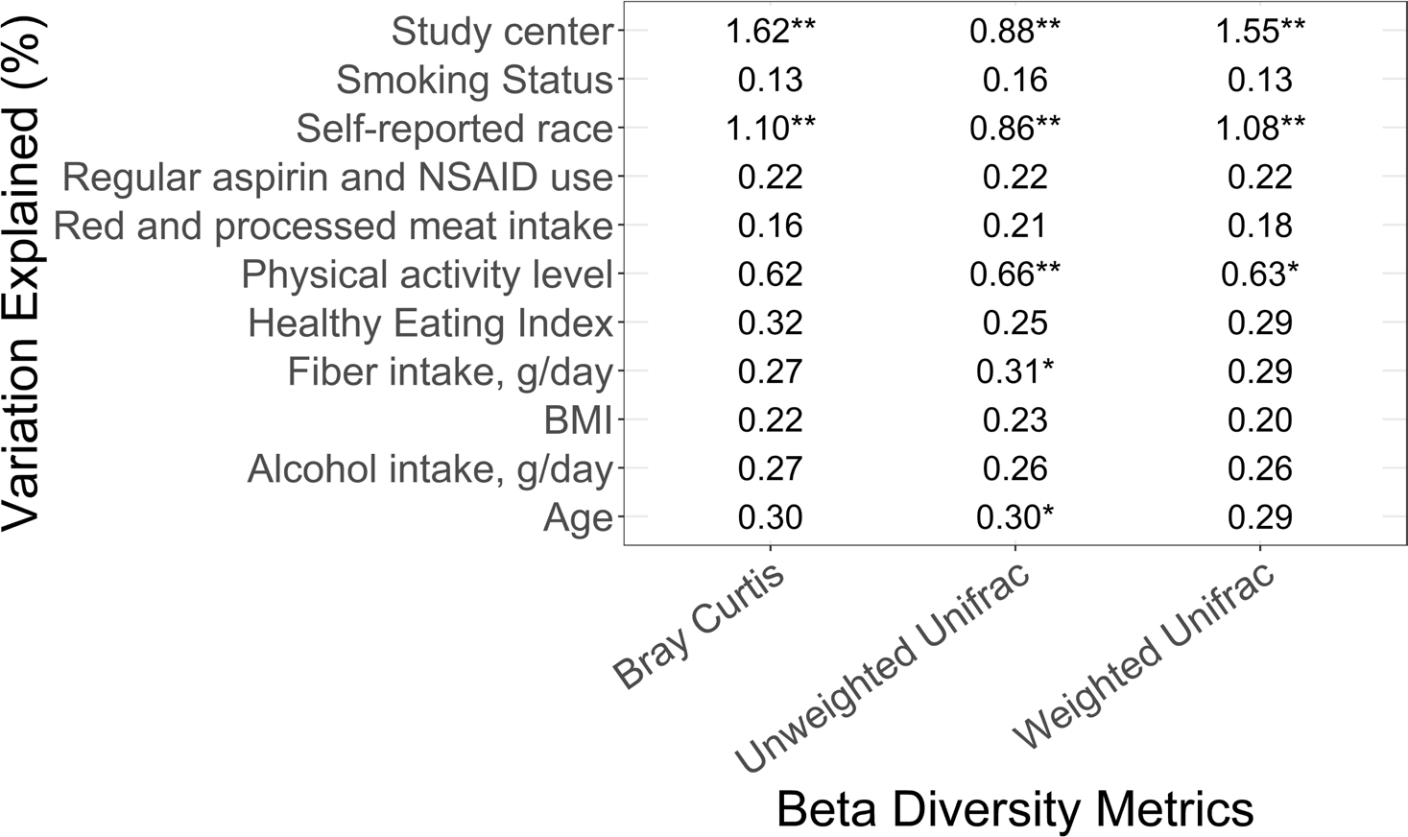
Percentage variations explained by race (White, Black, and other) and other factors in beta diversity Bray–Curtis, Weighted UniFrac, and Unweighted UniFrac distance matrices tested using the Adonis PERMANOVA test with 99 permutations. BMI: body mass index.

Race explained a statistically significant percentage of variation in the beta diversity matrices, including 1.10%, 0.86%, and 1.08% of the variability in Bray–Curtis, Unweighted Unifrac, and Weighted Unifrac distance matrices, respectively (Figure 1). The only measured factor explaining more variation than race was study center (1.62%, 0.88%, and 1.55%, respectively).

Colon tissue alpha diversity was not associated with adenoma presence. All associations were generally close to null (Table S5). These associations did not differ by adenoma characteristics, such as location, size, or grade of dysplasia (all *p*‐heterogeneity > 0.05). In MiRKAT tests assessing multivariate differences in overall microbiome composition, no beta diversity associations with adenoma were statistically significant (all *p* > 0.05; Table S6).

**FIGURE 2:**
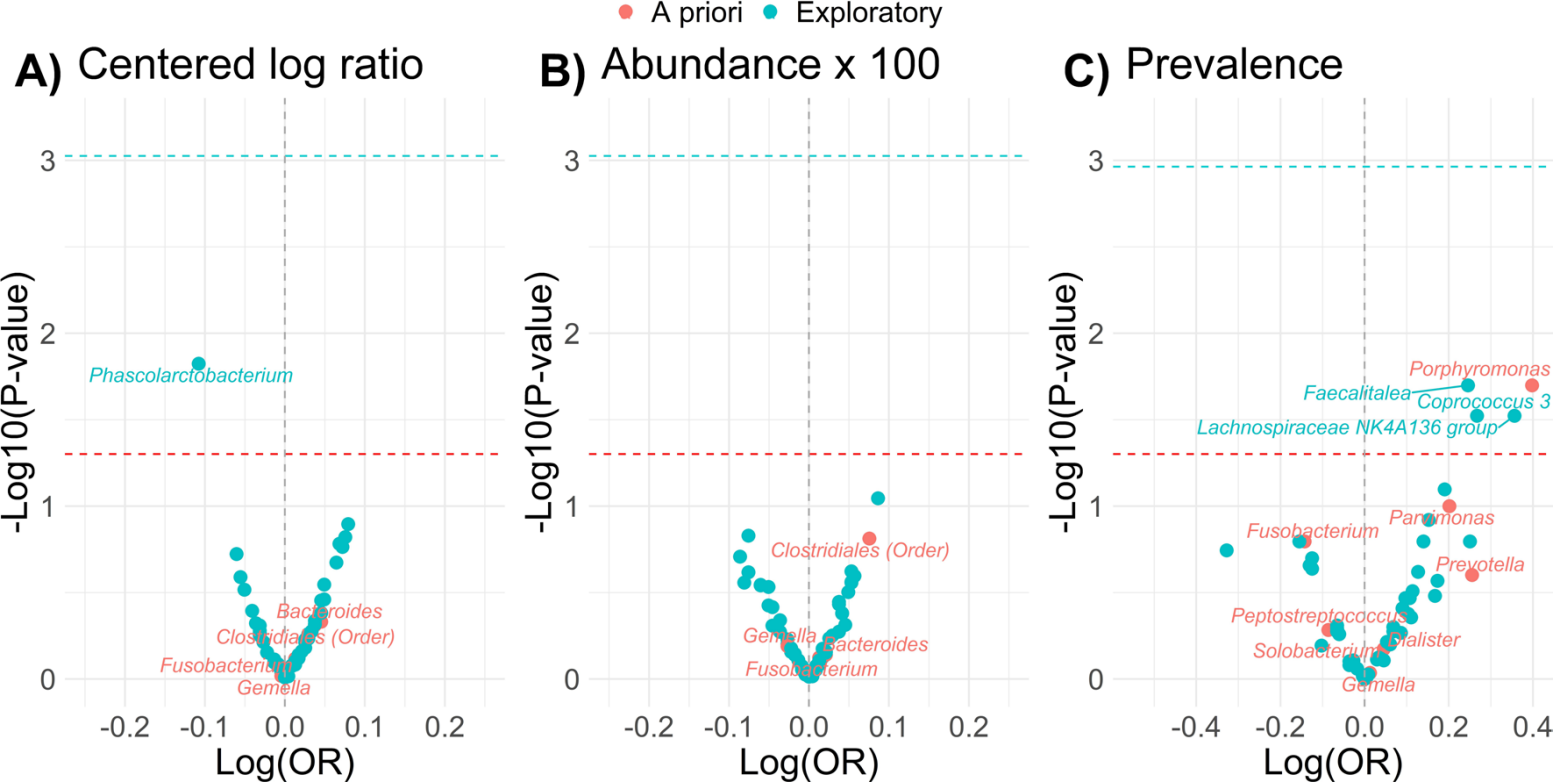
Volcano plot of the associations of bacteria with adenoma among *N* = 165 adenoma cases and *N* = 311 controls in the Colorectal Neoplasia Screening with Colonoscopy in Average‐Risk Women Regional Navy/Army Medical Centers study, 2000–2002; bacteria abundances were characterized using the (A) standardized centered log ratio transformation, (B) standardized relative abundance x 100, or (C) presence/absence of the bacterium; conditional logistic regression models adjusted for age, year of colonoscopy, regular nonsteroidal anti‐inflammatory drug and/or aspirin use (yes/no), current alcohol use (yes/no), family history of polyps (yes/no), education (high school education or less; or some college or more), hormone replacement therapy use (yes/no), daily fiber intake (g/day), body mass index (kg/m^2^), total daily energy intake (kcal/day), physical activity level (no moderate or vigorous activity, unknown, missing; moderate activity, or vigorous activity), race (Black, White, other), smoking status (current/former smokers or never smokers), red and processed meat intake (g/day), and study center (Walter Reed, San Diego, Portsmouth, or Bethesda); all bacteria are at the genus level except for order *Clostridiales*; we used Bonferroni correction to account for multiple testing the number of statistical comparisons (i.e., alpha level = 0.05/53 comparisons for abundance and 0.05/46 for prevalence) for all exploratory analyses (blue dotted line) and an alpha level of *p* = 0.05 for the a priori analysis (red dotted line).

As shown in Figure 2, in our exploratory analysis, the relative abundance of *Phascolarctobacterium* was most strongly, inversely associated with adenoma (OR = 0.78, 95% CI = 0.64, 0.95; *p* = 0.02) though this finding did not meet the Bonferroni threshold (see Table S7 for all ORs and 95% CIs). No relative abundances of other bacteria meeting the threshold for inclusion in our study were statistically significantly associated with colorectal adenomas. In our a priori presence/absence analyses, the presence of *Porphyromonas* (OR = 2.50, 95% CI = 1.18, 5.30; *p* = 0.02) was positively associated with colorectal adenomas. *Parvimonas* was also positively associated with adenomas, but more weakly so (OR *=* 1.59, 95% CI = 0.91, 2.78; *p* = 0.10). The presence of *Coprococcus* 3, *Faecalitalea*, and *Lachnospiraceae NK4A136 group* was also positively associated with adenomas. No microbial guilds (CAGs) were associated with adenomas after controlling for confounders (Table S8).

**FIGURE 3:**
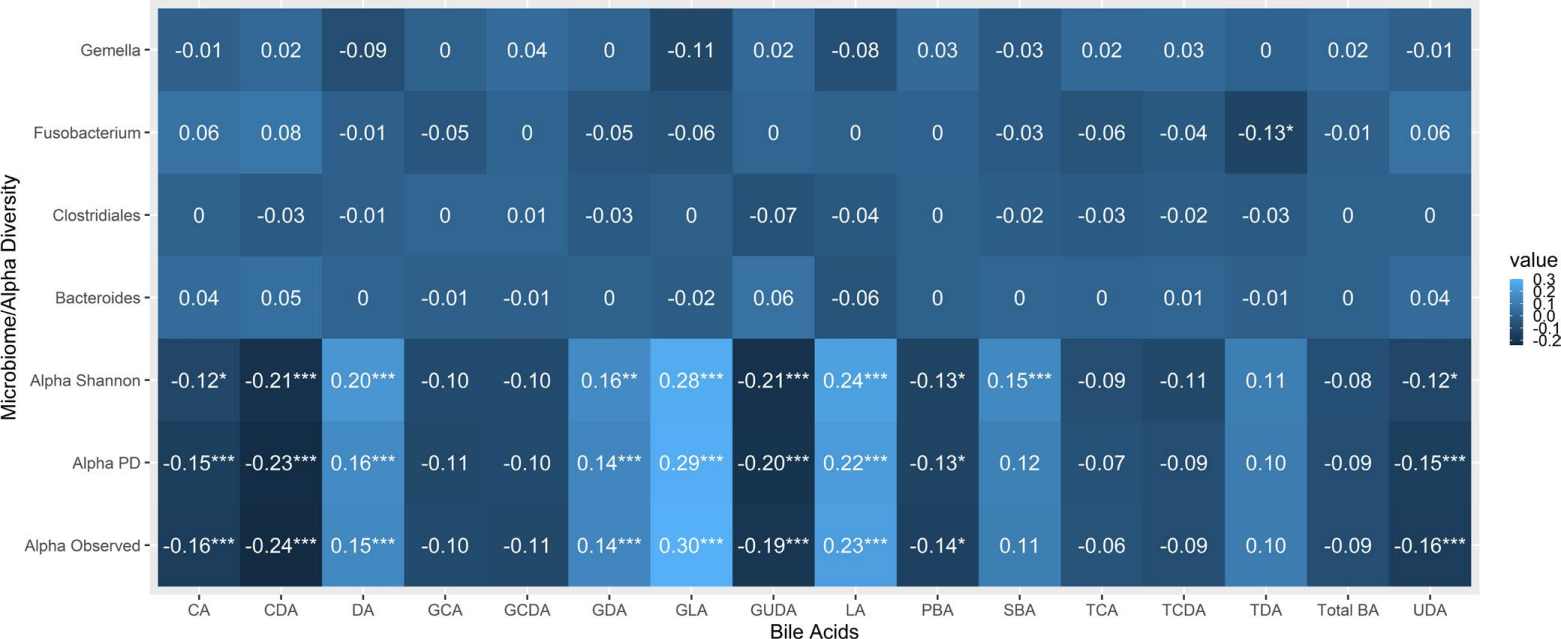
Partial Pearson correlations between the colon tissue microbiome and bile acids among *N* = 154 adenoma cases and *N* = 293 controls in the Colorectal Neoplasia Screening with Colonoscopy in Average‐Risk Women Regional Navy/Army Medical Centers study, 2000–2002; correlations were adjusted for age, year of colonoscopy, regular nonsteroidal anti‐inflammatory drug and/or aspirin use (yes/no), current alcohol use (yes/no), family history of polyps (yes/no), education (high school education or less; or some college or more), hormone replacement therapy use (yes/no), daily fiber intake (g/day), body mass index (kg/m^2^), total daily energy intake (kcal/day), physical activity level (no moderate or vigorous activity, unknown, missing; moderate activity, or vigorous activity), race (Black, White, other), smoking status (current/former smokers or never smokers), red and processed meat intake (g/day), and study center (Walter Reed, San Diego, Portsmouth, or Bethesda).***p < 0.001; **p < 0.01; *p < 0.05. Relative abundances are multiplied by 100 and standardized by the control's distribution. *p* values are corrected for multiple testing using the Benjamini–Hochberg method. CA, cholate; CDA, chenodeoxycholate; DA, deoxycholate, GCA, glycocholate; GCDA, glycochenodeoxycholate; GDA, glycodeoxycholate; GLA, glycolithocholate; GUDA, glycoursodeoxycholate; LA, lithocholate; PBA, primary bile acids; PD, Faith's phylogeny; SBA, secondary bile acids; TCA, taurocholate; TCDA, taurochenodeoxycholate; TDA, taurodeoxycholate; UDA, ursodeoxycholate.

Circulating concentrations of BAs were generally positively but not statistically significantly associated with adenomas (Table S9). For example, comparing those in the highest relative to lowest tertile of total BAs, there were 39% higher odds of colorectal adenomas (95% CI = 0.82, 2.36; *p*‐trend = 0.10) though these findings were not statistically significant. The findings were generally similar across adenoma characteristics, including anatomical location. Alpha diversity was most strongly positively associated with glycolithocholic acid, lithocholic acid, and deoxycholic acid, and most negatively associated with chenodeoxycholic acid, glycoursodeoxycholic acid, and ursodeoxycholic acid. Glycolithocholic acid was positively associated with *Phascolarctobacterium* (Figure 3).

## 4 Discussion

In this colonoscopy‐based case–control study among women at average risk for CRC, we found that overall microbiome composition differed across race and geography. Normal colon tissue alpha and beta diversity were not associated with colorectal adenoma; however, the presence of a priori bacteria of oral origin was positively associated with adenoma presence. We also found that circulating BA concentrations were positively, albeit somewhat weakly, associated with adenoma presence. Our findings reflect the potential for some microbially related mechanisms underlying the incidence of CRC among women, though this should be explored in larger populations.

We found that there were differences in the overall microbiome composition between Black women and White women, similar to the growing literature suggesting gut microbiome differences by race [24, 25]. Exposures related to social determinants of health—encompassing psychosocial, socioeconomic, cultural, dietary/lifestyle (e.g., access to nutritious food), and healthcare/policy‐related domains—likely interact to influence biology, including potentially the colon epithelial microbiome [26, 27]. At present, there are few studies that have compared the tissue microbiome across racial groups. In one study [28], which included 97 Black and 56 non‐Hispanic White CRC cases, and 100 Black and 76 non‐Hispanic White controls, there were race‐specific associations of uninvolved/normal colonic tissue microbes with CRC. Taken together, studies with large enough sample sizes of diverse individuals are needed to conduct more in‐depth investigations into the gut microbiome within strata of higher risk population groups.

There is growing plausibility for a role of gut colonizing bacteria in colorectal carcinogenesis. We did not find any notable differences with regard to alpha or beta diversity, unlike a previous study which noted beta diversity differences in the normal colon tissue from 21 individuals with polyps compared to 56 healthy controls [29]. We found that the presence of *Porphyromonas—*and more weakly *Parvimonas*—was positively associated with adenoma prevalence. Oral originating microbes have been positively associated with colorectal neoplasms in multiple previous studies. For example, in a meta‐analysis of four case–control studies comparing 16S ribosomal RNA (rRNA) gene sequencing data from CRC case tumor tissue and normal tissue from healthy controls, the relative abundance of *Parvimonas micra* was higher among the CRC tumor samples [5]. In multiple additional case–control studies comparing CRC cases to healthy controls, the abundance of *Parvimonas* and *Porphyromonas* was higher in CRC case tumor tissue compared to controls [4, 30, 31, 32]. In one of the larger studies among *N* = 47 cases with paired adenoma and adenoma adjacent tissue, *N* = 52 with paired colorectal carcinoma and carcinoma adjacent tissue, and *N* = 61 healthy controls, in univariate analyses, there were no differences in *Porphyromonas* or *Parvimonas* between adenoma nor adenoma‐adjacent mucosal tissue compared to healthy control mucosal tissue. However, the abundance of these bacteria among colorectal carcinoma‐ and carcinoma‐adjacent tissue was statistically significantly higher than adenoma/adenoma‐adjacent tissue and healthy control tissue [33]. Multiple factors could influence differences in our findings compared to prior findings, including sampling site (e.g., rectal, colon, or neoplasm) which may reflect different scientific questions. For example, normal tissue may better reflect the microbial community that preceded the development of the neoplasm, whereas neoplasm tissue may more so reflect the microbial community that colonized the tissue once the neoplasm occurred. It is possible that oral bacteria could have different associations with precursors of CRC than colorectal tumors [34]. There may also be differences due to sex (our study only includes women) or other demographic factors.

Gut microbes contain the potential to modify and produce metabolically active molecules that may influence colorectal carcinogenesis, including BAs. We found that BAs were positively associated with adenoma prevalence, though the associations were not statistically significant. Multiple prior studies, both cross‐sectional and prospective, have suggested positive associations of circulating BA concentrations with colorectal neoplasms [35, 36, 37, 38, 39], including a study from our group. We previously investigated associations of BAs with adenoma recurrence in the prospective Polyp Prevention Trial [40] among *N* = 129 individuals with an adenoma recurrence and *N* = 239 individuals without a recurrence. In this study, we measured circulating BAs at the time of the initial, baseline adenoma diagnosis and at 2 and 3 years after adenoma diagnosis and estimated associations of BAs at each timepoint with adenoma recurrence 4 years after diagnosis. Intriguingly, we found that the associations of BAs (mostly primary BAs) with adenoma recurrence were generally strongest for associations of baseline BAs (i.e., approximately 4 years prior to recurrence) rather than year 2 and year 3 (which were closer to adenoma diagnosis). Overall, there seems to be growing support for a role of BAs in colorectal carcinogenesis; however, our findings did not strongly support a role of BAs in association with prevalent CRC precursors. Differences in findings could be due to study population differences and the temporality of the measurements (i.e., our estimated associations of BAs with adenoma were cross‐sectional).

Our study is among the largest studies of the colon tissue microbiome and adenoma to date, with the largest population of women. There are also some limitations of our study. Despite our sample size, we still had limited power to evaluate associations across all bacteria or interactions between bacteria and circulating BAs. There is also the possibility of chance findings; however, we primarily focused on a priori‐selected bacteria and included multiple testing correction to avoid false positive findings in our exploratory analyses. Further, we were unable to evaluate the functional capacity of the bacteria in relation to adenoma presence due to our use of 16S rRNA gene sequencing. In addition, we only measured circulating BA concentrations, which are moderately to weakly correlated with fecal BA concentrations, the latter of which may be more relevant for CRC development [41]. Finally, we leveraged colon tissue from the normal epithelium, which may not reflect the microbial communities of the full colon and rectum and likely has a different microbial composition than tumor tissue or fecal samples that have been more frequently studied; however, normal tissue colon biopsies may be a useful specimen to characterize the bacteria that invade the epithelial crypts, perhaps being more likely to have influences on CRC development. Our findings were based on a case–control study and, like most of the microbiome–cancer literature, were cross‐sectional in nature. We previously found that associations of the tissue microbiome with adenomas could be stronger when considered cross‐sectionally, rather than prospectively [14]. Future studies should include longitudinal assessments to elucidate the prospective role of the microbiome in CRC risk. This longitudinal design would also address the potential for reverse causality in the associations observed (i.e., whether the neoplasm led to differences in the microbiome or vice versa).

Taken together, our findings suggest that certain oral‐originating bacteria are positively associated with adenoma presence. Our findings supported a potentially positive, but weaker, association of BAs with adenomas. Further, our results highlight the need for large, population‐based studies that investigate colorectal neoplasm associations among diverse populations with detailed data on modifiable and non‐modifiable exposures. Ultimately, continued research in this area may highlight potential methods to intervene on the gut microbiome to reduce the population burden of CRC.

## Author Contributions

**Casey Dagnall:** conceptualization, methodology, writing – review and editing, writing – original draft. **Doratha A. Byrd:** conceptualization, writing – original draft, writing – review and editing, methodology, formal analysis, supervision. **Emily Vogtmann:** conceptualization, methodology, writing – original draft, writing – review and editing, formal analysis, supervision. **Erikka Loftfield:** conceptualization, methodology, writing – original draft, writing – review and editing. **Belynda Hicks:** conceptualization, methodology, writing – review and editing, writing – original draft. **Jessica R. Burns:** writing – original draft, writing – review and editing, formal analysis. **Jianxin Shi:** supervision, conceptualization, methodology, writing – review and editing, writing – original draft, formal analysis. **Jin Xu:** formal analysis, writing – original draft, writing – review and editing. **Joshua Sampson:** conceptualization, methodology, writing – original draft, writing – review and editing. **Kristine Jones:** conceptualization, methodology, writing – review and editing, writing – original draft. **Maria F. Gomez:** formal analysis, writing – review and editing, writing – original draft. **Nate Smith:** writing – original draft, writing – review and editing, formal analysis. **Rashmi Sinha:** formal analysis, conceptualization, methodology, writing – original draft, writing – review and editing, supervision. **Stephanie R. Hogue:** formal analysis, writing – original draft, writing – review and editing. **Andrew Warner:** conceptualization, methodology, writing – original draft, writing – review and editing. **Patricia G. Wolf:** conceptualization, methodology, writing – original draft, writing – review and editing. **Youngchul Kim:** formal analysis, writing – review and editing, writing – original draft. **Yunhu Wan:** conceptualization, methodology, writing – review and editing, writing – original draft.

## Conflicts of Interest

The authors declare no conflicts of interest.

## Supporting information


Data S1.

## Data Availability

The 16s rRNA gene amplicon sequencing data are publically available in the National Center for Biotechnology (NCBI) Sequence Read Archive (https://www.ncbi.nlm.nih.gov/sra) under the accession number SRA: PRJNA1068138. Clinical data used for analysis are available upon request. The code used for the bioinformatics analysis and statistics is available upon request.
