# Supplementary material for: Associations of the Colon Tissue Microbiome and Circulating Bile Acids With Colorectal Adenoma Among Average‐Risk Women
Source: Cancer Med. 2025 Sep 12;14(18):e71048. doi: 10.1002/cam4.71048 (PMC12426762; doi:10.1002/cam4.71048)
Supplement: Supplementary file 1 — Data S1. [file CAM4-14-e71048-s001.docx]

**Supplemental Tables and Figures**

**Supplemental Figure 1.** Principal coordinate analysis plots of (A) Bray Curtis, (B) Weighted UniFrac, and (C)

Unweighted UniFrac distances of quality control samples (n= 16 artificial community, n=10 extraction

blanks, and n=16 robogut) and cases (n= 258) and control (n=638) in the Colonoscopy in Average-Risk

Women Regional Navy/Army Medical Centers study, 2000-2002


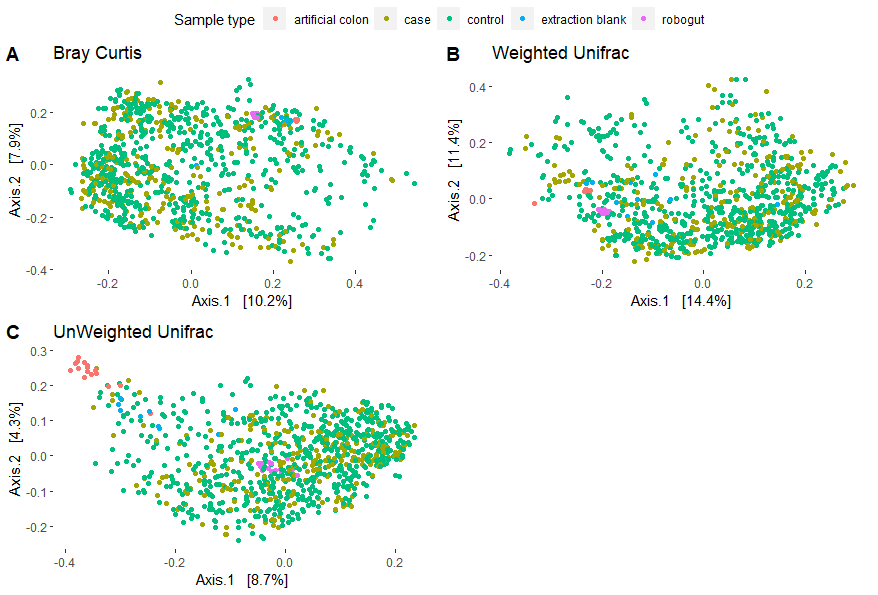


**Supplemental Table 1.** Coefficients of variation for microbiome metrics among N=42 QC samples

| Alpha.Diversity | Artificial community, N=22 | Robogut, N=20 |
| --- | --- | --- |
| Observed ASVs | 17.03 | 9.58 |
| Shannon Index | 1.48 | 1.81 |
| Faith’s PD | 16.30 | 8.29 |

**Supplemental Table 2.** Number of taxa and samples removed at different rarefaction sequencing reads depth for QC and patient samples

| Reads depth | Number of taxa | Number of samples | Taxa removed | Samples removed |
| --- | --- | --- | --- | --- |
| Original | 9,456 | 938 | 0 | 0 |
| 11 | 905 | 938 | 8,551 | 0 |
| 500 | 4,187 | 932 | 5,269 | 6 |
| 2000 | 5,910 | 923 | 3,546 | 15 |
| 5000 | 7,160 | 906 | 2,296 | 32 |
| 11000 | 8,065 | 844 | 1,391 | 94 |
| 15000 | 8,382 | 848 | 1,074 | 90 |
| 17000 | 8,466 | 836 | 990 | 102 |

**Supplemental Figure 2.** Rarefaction curves for (A) Observed ASV and (B) Shannon alpha diversity indices for

quality control samples (n= 16 artificial community, n=10 extraction blanks, and n=16 robogut) and cases

(n= 258) and control (n=638) samples in the Colonoscopy in Average-Risk Women Regional Navy/Army

Medical Centers study, 2000-2002


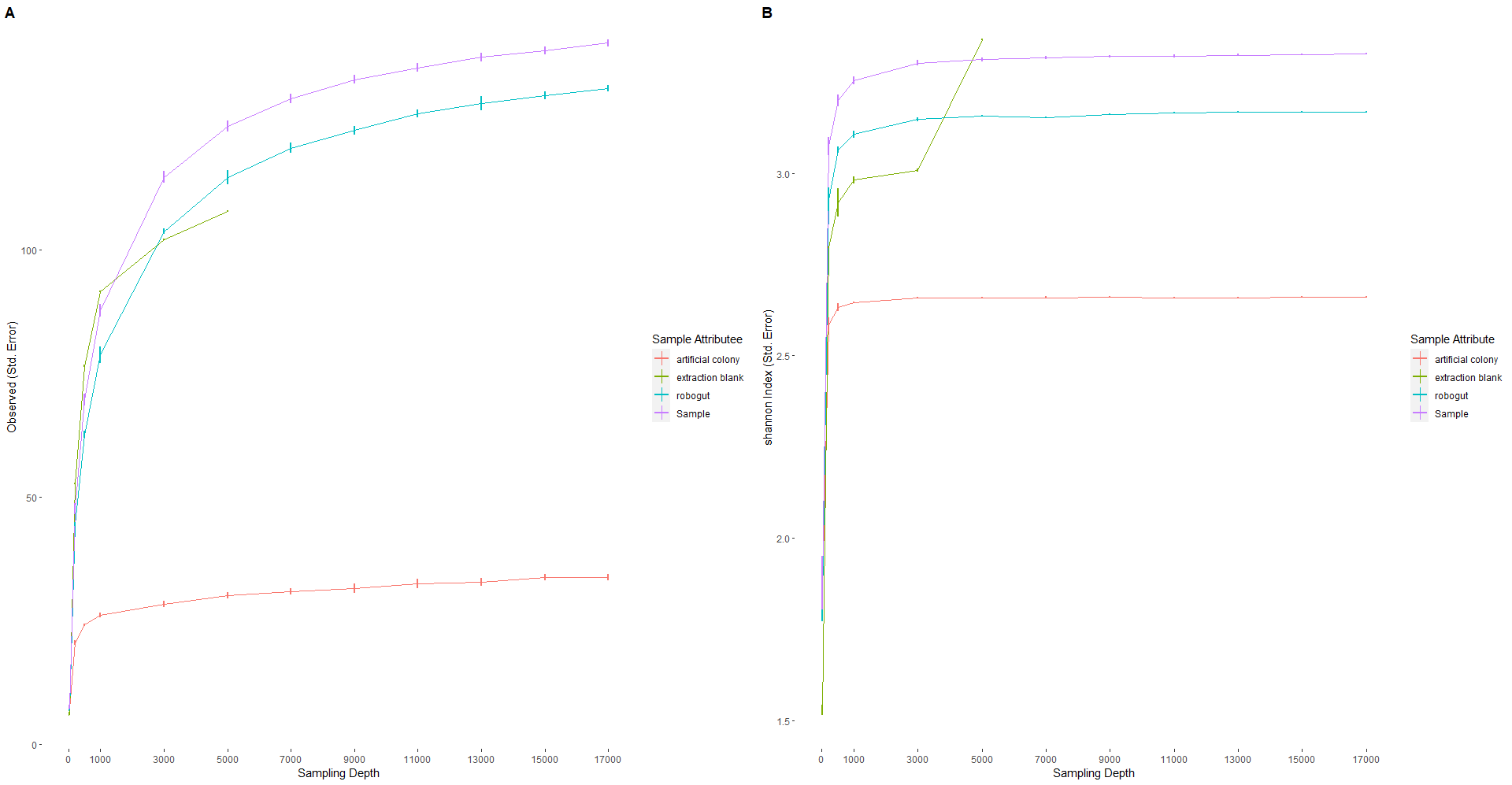


**Supplemental Table 3.** Intraclass correlation coefficients for N=50 tissue pairs taken from the splenic flexure

| **Microbiome metric** | **ICC (95% CI)** |
| --- | --- |
| **Alpha Diversity** |  |
| Shannon Index | 0.92 (0.90, 0.94) |
| Observed ASVs | 0.95 (0.94, 0.96) |
| Faith’s PD | 0.92 (0.90, 0.93) |
| ***A priori-*selected bacteria, relative abundance** |  |
| *Bacteroides* | 0.86 (0.83, 0.88) |
| *Fusobacterium* | 0.96 (0.95, 0.96) |
| *Gemella* | 0.88 (0.85, 0.90) |
| Clostridiales (Order) | 0.88 (0.85, 0.90) |

Abbreviations: ICC, intraclass correlation coefficient

**Supplemental Table 4.** Coefficients of variation for bile acid (BA) concentrations among N=24 QC samples

| Bile Acid, ng/mL | Pooled QC Serum 1 | Pooled QC Serum 2 |
| --- | --- | --- |
| Chenodeoxycholic | 1.56 | 2.5 |
| Cholic | 3.95 | 1.64 |
| Deoxycholic | 5.47 | 2.86 |
| Glycochenodeoxycholic | 3.82 | 5.39 |
| Glycocholic | 9.96 | 8.76 |
| Glycodeoxycholic | 4.93 | 2.38 |
| Glycolithocholic | 4.71 | 3.68 |
| Glycoursodeoxycholic | 11.52 | 9.38 |
| Lithocholic | 7.41 | 8.69 |
| Taurochenodeoxycholic | 8.04 | 6.26 |
| Taurocholic | 4.37 | 11.71 |
| Taurodeoxycholic | 7.7 | 6.98 |
| Taurolithocholic | 10.15 | 18.46 |
| Tauroursodeoxycholic | 20.08 | 40.06 |
| Ursodeoxycholic | 4.56 | 3.71 |

Abbreviations: QC, quality control

**Supplemental Table 5.** Associations^a^ of colon tissue alpha diversity with colorectal adenoma among N=165 adenoma cases and N=311 controls in the Colorectal Neoplasia Screening with Colonoscopy in Average-Risk Women Regional Navy/Army Medical Centers study, 2000-2002

|  |  |  |  | **Adenoma characteristics** | | | |
| --- | --- | --- | --- | --- | --- | --- | --- |
|  |  |  | **Overall** | **Advanced ^b^** | **Non-advanced** | **Distal ^c^** | **Proximal ^d^** |
| **Alpha Diversity Metric** | **Range** | **No. cases/control** | **OR (95% CI)** | | | | |
| **Shannon Index** |  |  |  |  |  |  |  |
| Continuous |  |  | 0.92 (0.74, 1.15) | 0.93 (0.71, 1.23) | 1.03 (0.80, 1.32) | 0.94 (0.72, 1.23) | 1.01 (0.79, 1.30) |
| T1 | (1.56, 3.19) | 53/103 | 1.00 | 1.00 | 1.00 | 1.00 | 1.00 |
| T2 | (3.20, 3.65) | 56/102 | 0.88 (0.52, 1.50) | 0.87 (0.44, 1.71) | 1.13 (0.63, 2.03) | 0.79 (0.41, 1.55) | 1.18 (0.65, 2.15) |
| T3 | (3.65, 4.72) | 56/106 | 0.92 (0.53, 1.59) | 0.87 (0.45, 1.69) | 1.14 (0.63, 2.07) | 0.86 (0.45, 1.65) | 1.12 (0.61, 2.06) |
| *P-trend* |  |  | *0.76* | *0.59* | *0.59* | *0.57* | *0.66* |
| *P-heterogeneity^e^* |  |  |  | *0.41* | *Ref.* | *0.82* | *Ref.* |
| **Observed ASV** |  |  |  |  |  |  |  |
| Continuous |  |  | 0.99 (0.80, 1.22) | 0.98 (0.74, 1.29) | 1.11 (0.88, 1.41) | 1.02 (0.77, 1.34) | 1.07 (0.84, 1.37) |
| T1 | (28.60, 110.30) | 49/104 | 1.00 | 1.00 | 1.00 | 1.00 | 1.00 |
| T2 | (110.40, 145.30) | 58/101 | 1.21 (0.70, 2.10) | 1.18 (0.61, 2.29) | 1.48 (0.81, 2.72) | 0.98 (0.49, 1.94) | 1.63 (0.88, 3.01) |
| T3 | (145.50, 335.60) | 58/106 | 1.07 (0.62, 1.82) | 0.87 (0.44, 1.72) | 1.43 (0.79, 2.62) | 1.08 (0.56, 2.06) | 1.22 (0.65, 2.28) |
| *P-trend* |  |  | *0.92* | *0.63* | *0.28* | *0.81* | *0.66* |
| *P-heterogeneity^e^* |  |  |  | *0.27* | *Ref.* | *0.76* | *Ref.* |
| **Faith's PD** |  |  |  |  |  |  |  |
| Continuous |  |  | 1.00 (0.81, 1.24) | 0.99 (0.75, 1.31) | 1.12 (0.88, 1.43) | 1.04 (0.79, 1.37) | 1.07 (0.84, 1.37) |
| T1 | (167.14, 501.31) | 49/103 | 1.00 | 1.00 | 1.00 | 1.00 | 1.00 |
| T2 | (501.48, 624.60) | 58/102 | 1.21 (0.69, 2.12) | 1.13 (0.57, 2.24) | 1.48 (0.80, 2.72) | 1.02 (0.51, 2.01) | 1.54 (0.83, 2.86) |
| T3 | (625.35, 1151.77) | 58/106 | 1.03 (0.60, 1.77) | 0.91 (0.46, 1.79) | 1.38 (0.75, 2.52) | 1.01 (0.52, 1.94) | 1.27 (0.68, 2.35) |
| *P-trend* |  |  | *0.99* | *0.73* | *0.32* | *0.99* | *0.52* |
| *P-heterogeneity^e^* |  |  |  | *0.28* | *Ref.* | *0.87* | *Ref.* |

Abbreviations: ASV, amplicon sequence variants; PD, phylogenetic diversity

^a^ Odds ratios and 95% confidence intervals were estimated using conditional logistic regression (overall associations) and multinomial logistic regression (by adenoma characteristics) and were adjusted for age, year of colonoscopy, regular non-steroidal anti-inflammatory drug and/or aspirin use (yes/no), current alcohol use (yes/no), family history of polyps (yes/no), education (high school education or less; or some college or more), hormone replacement therapy use (yes/no), daily fiber intake (g/day), body mass index (kg/m^2^), total daily energy intake (kcal/day), physical activity level (no moderate or vigorous activity, unknown, missing; moderate activity, or vigorous activity), race (Black, White, other), smoking status (current/former smokers or never smokers), red and processed meat intake (g/day), and study center (Walter Reed, San Diego, Portsmouth, or Bethesda).

^b^ Advanced adenomas are defined as having ≥ 1 adenoma with a villous component, ≥ 1 cm diameter, or high grade dysplasia, and/or having multiple adenomas

^c^ Distal adenomas were defined as the most advanced adenoma being in the splenic flexure, descending, or sigmoid colon

^d^ Proximal adenomas were defined as the most advanced adenoma being in the cecum, ascending, hepatic flexure, or transverse colon

^e^ To calculate the P-heterogeneity, we conducted a case-only multivariable logistic regression analysis with adenoma characteristic as the dependent variable, and took the *P* value for the continuous alpha diversity metric to be the *P*_heterogeneity_.Non-advanced adenomas served as the reference for advanced adenomas, proximal was the reference for distal adenomas.

**Supplemental Table 6.** P-values^a^ from microbiome regression-based analyses for Bray Curtis, Unweighted UniFrac, and Weighted UniFact Beta diversity distance matrices

| Test | Bray Curtis | Unweighted Unifrac | Weighted Unifrac | Omnibus |
| --- | --- | --- | --- | --- |
| Davies | 0.896 | 0.999 | 0.873 | 0.979 |
| Permutation | 0.897 | 0.999 | 0.871 | 0.971 |

^a^ P-value estimates are adjusted for age, year of colonoscopy, regular non-steroidal anti-inflammatory drug and/or aspirin use (yes/no), current alcohol use (yes/no), family history of polyps (yes/no), education (high school education or less; or some college or more), hormone replacement therapy use (yes/no), daily fiber intake (g/day), body mass index (kg/m^2^), total daily energy intake (kcal/day), physical activity level (no moderate or vigorous activity, unknown, missing; moderate activity, or vigorous activity), race (Black, White, other), smoking status (current/former smokers or never smokers), red and processed meat intake (g/day), and study center (Walter Reed, San Diego, Portsmouth, or Bethesda)

**Supplemental Table 7.** Associations^a^ of colon tissue bacterial abundance and prevalence with colorectal adenoma among N=165 adenoma cases and N=311 controls in the Colorectal Neoplasia Screening with Colonoscopy in Average-Risk Women Regional Navy/Army Medical Centers study, 2000-2002

| **Relative Abundance** |  | |  | CLR Transformed^b^ | |  | Abundance x 100^c^ | |  | Prevalence^d^ | | | |
| --- | --- | --- | --- | --- | --- | --- | --- | --- | --- | --- | --- | --- | --- |
|  | Cases, N= 165 | Controls, N=311 |  | OR (95% CI) CLR | P-value |  | OR (95% CI) | P-value |  | Cases, N= 165 | Controls, N=311 | OR (95% CI) | P-value |
|  | Median (min-max) | Median (min-max) |  |  |  |  |  |  |  | Prevalent N (%) | Prevalent N (%) |  |  |
| *A priori*-selected bacteria |  |  |  |  |  |  |  |  |  |  |  |  |  |
| *Bacteroides* | 26.73 (0.02, 72.29) | 26.090 (0.00, 85.72) |  | 1.11 (0.84, 1.46) | 0.47 |  | 1.05 (0.81, 1.34) | 0.72 |  | 165 (100) | 309 (99) | - | - |
| *Clostridiales* | 59.38 (9.96, 87.49) | 57.393 (0.75, 91.63) |  | 1.09 (0.85, 1.40) | 0.49 |  | 1.19 (0.94, 1.50) | 0.15 |  | 165 (100) | 311 (100) | - | - |
| *Dialister* | 0.00 (0.00, 3.51) | 0.000 (0.00, 3.84) |  | - | - |  | - | - |  | 55 (33) | 92 (30) | 1.11 (0.69, 1.80) | 0.67 |
| *Fusobacterium* | 0.01 (0.00, 37.37) | 0.011 (0.00, 66.26) |  | 1.03 (0.83, 1.29) | 0.76 |  | 1.03 (0.84, 1.27) | 0.75 |  | 85 (52) | 167 (54) | 0.72 (0.46, 1.13) | 0.16 |
| *Gemella* | 0.05 (0.00, 3.23) | 0.045 (0.00, 5.42) |  | 0.99 (0.79, 1.25) | 0.96 |  | 0.94 (0.71, 1.24) | 0.65 |  | 134 (81) | 249 (80) | 1.03 (0.59, 1.80) | 0.92 |
| *Parvimonas* | 0.00 (0.00, 0.30) | 0.000 (0.00, 1.16) |  | - | - |  | - | - |  | 40 (24) | 55 (18) | 1.59 (0.91, 2.78) | 0.10 |
| *Peptostreptococcus* | 0.00 (0.00, 0.40) | 0.000 (0.00, 1.00) |  | - | - |  | - | - |  | 29 (18) | 53 (17) | 0.82 (0.44, 1.50) | 0.52 |
| *Porphyromonas* | 0.00 (0.00, 0.53) | 0.000 (0.00, 0.49) |  | - | - |  | - | - |  | 25 (15) | 21 (7) | 2.50 (1.18, 5.30) | 0.02 |
| *Prevotella* | 0.00 (0.00, 0.21) | 0.000 (0.00, 0.07) |  | - | - |  | - | - |  | 11 (6) | 13 (4) | 1.80 (0.66, 4.93) | 0.25 |
| *Solobacterium* | 0.00 (0.00, 0.37) | 0.000 (0.00, 0.62) |  | - | - |  | - | - |  | 36 (22) | 75 (24) | 0.94 (0.57, 1.54) | 0.79 |
| Exploratory-selected bacteria |  |  |  |  |  |  |  |  |  |  |  |  |  |
| *[Clostridium] innocuum group* | 0.03 (0.00, 2.27) | 0.029 (0.00, 2.95) |  | 0.94 (0.75, 1.18) | 0.61 |  | 1.04 (0.82, 1.31) | 0.75 |  | 110 (67) | 202 (65) | 1.17 (0.74, 1.83) | 0.50 |
| *[Eubacterium] coprostanoligenes group* | 0.01 (0.00, 1.87) | 0.009 (0.00, 2.72) |  | 0.91 (0.73, 1.13) | 0.40 |  | 0.84 (0.67, 1.06) | 0.15 |  | 92 (56) | 164 (53) | 1.13 (0.72, 1.77) | 0.61 |
| *[Eubacterium] hallii group* | 0.38 (0.00, 6.46) | 0.325 (0.00, 4.62) |  | 1.12 (0.89, 1.40) | 0.35 |  | 1.06 (0.86, 1.31) | 0.58 |  | 147 (89) | 262 (84) | 1.49 (0.74, 3.00) | 0.27 |
| *[Ruminococcus] gauvreauii group* | 0.05 (0.00, 1.41) | 0.056 (0.00, 3.76) |  | 1.08 (0.84, 1.40) | 0.53 |  | 0.95 (0.75, 1.22) | 0.70 |  | 101 (61) | 196 (63) | 0.87 (0.56, 1.36) | 0.55 |
| *[Ruminococcus] gnavus group* | 1.31 (0.00, 52.91) | 1.044 (0.00, 56.08) |  | 1.18 (0.93, 1.50) | 0.17 |  | 1.14 (0.91, 1.41) | 0.25 |  | 137 (83) | 260 (84) | 1.02 (0.56, 1.85) | 0.94 |
| *[Ruminococcus] torques group* | 8.23 (0.00, 40.24) | 7.346 (0.00, 55.48) |  | 1.06 (0.84, 1.35) | 0.61 |  | 1.10 (0.88, 1.37) | 0.42 |  | 162 (98) | 307 (99) | - | - |
| *Agathobacter* | 0.77 (0.00, 12.17) | 0.700 (0.00, 20.97) |  | 1.06 (0.85, 1.32) | 0.59 |  | 0.94 (0.74, 1.19) | 0.59 |  | 135 (82) | 246 (79) | 1.09 (0.62, 1.93) | 0.76 |
| *Akkermansia* | 0.01 (0.00, 16.51) | 0.025 (0.00, 26.60) |  | 0.93 (0.75, 1.14) | 0.49 |  | 0.92 (0.72, 1.19) | 0.53 |  | 93 (56) | 182 (59) | 0.75 (0.47, 1.19) | 0.23 |
| *Alistipes* | 0.59 (0.00, 8.14) | 0.568 (0.00, 15.58) |  | 1.04 (0.83, 1.30) | 0.75 |  | 1.02 (0.82, 1.27) | 0.84 |  | 147 (89) | 280 (90) | 0.92 (0.43, 1.96) | 0.83 |
| *Anaerostipes* | 0.37 (0.00, 9.65) | 0.370 (0.00, 14.02) |  | 1.07 (0.85, 1.34) | 0.58 |  | 0.96 (0.75, 1.22) | 0.72 |  | 152 (92) | 298 (96) | - | - |
| *Bilophila* | 0.16 (0.00, 3.83) | 0.097 (0.00, 2.68) |  | 1.07 (0.86, 1.33) | 0.55 |  | 1.09 (0.91, 1.30) | 0.37 |  | 115 (70) | 200 (64) | 0.96 (0.60, 1.55) | 0.87 |
| *Blautia* | 3.48 (0.00, 18.91) | 3.438 (0.00, 17.11) |  | 0.93 (0.72, 1.18) | 0.54 |  | 0.87 (0.68, 1.12) | 0.29 |  | 164 (99) | 310 (99) | - | - |
| *Butyricicoccus* | 0.37 (0.00, 2.69) | 0.344 (0.00, 4.81) |  | 1.05 (0.83, 1.33) | 0.69 |  | 1.04 (0.84, 1.30) | 0.71 |  | 156 (95) | 295 (95) | 0.79 (0.30, 2.10) | 0.64 |
| *Christensenellaceae R-7 group* | 0.06 (0.00, 4.02) | 0.078 (0.00, 17.93) |  | 0.87 (0.71, 1.07) | 0.19 |  | 0.83 (0.59, 1.16) | 0.28 |  | 114 (69) | 205 (66) | 1.07 (0.66, 1.73) | 0.77 |
| *Collinsella* | 0.20 (0.00, 2.18) | 0.151 (0.00, 3.72) |  | 0.95 (0.75, 1.21) | 0.70 |  | 0.87 (0.67, 1.13) | 0.29 |  | 115 (70) | 211 (68) | 0.70 (0.43, 1.15) | 0.16 |
| *Coprococcus 1* | 0.59 (0.00, 4.78) | 0.257 (0.00, 8.10) |  | 1.16 (0.92, 1.45) | 0.21 |  | 1.05 (0.82, 1.33) | 0.70 |  | 109 (66) | 179 (58) | 1.38 (0.88, 2.18) | 0.16 |
| *Coprococcus 3* | 0.53 (0.00, 5.92) | 0.456 (0.00, 4.93) |  | 1.11 (0.87, 1.41) | 0.41 |  | 1.12 (0.90, 1.40) | 0.31 |  | 126 (76) | 204 (66) | 1.85 (1.07, 3.19) | 0.03 |
| *Dorea* | 3.42 (0.00, 13.91) | 3.121 (0.00, 14.19) |  | 1.19 (0.94, 1.50) | 0.15 |  | 1.22 (0.97, 1.52) | 0.09 |  | 153 (93) | 273 (88) | 1.78 (0.80, 3.96) | 0.16 |
| *Erysipelatoclostridium* | 0.37 (0.00, 28.68) | 0.348 (0.00, 25.70) |  | 1.03 (0.83, 1.28) | 0.76 |  | 1.01 (0.80, 1.26) | 0.96 |  | 144 (87) | 258 (83) | 1.22 (0.65, 2.29) | 0.54 |
| *Erysipelotrichaceae UCG-003* | 0.50 (0.00, 22.28) | 0.492 (0.00, 21.75) |  | 1.00 (0.80, 1.24) | 0.97 |  | 0.90 (0.70, 1.15) | 0.38 |  | 123 (75) | 209 (67) | 1.55 (0.94, 2.55) | 0.08 |
| *Faecalibacterium* | 9.18 (0.00, 25.93) | 8.966 (0.00, 23.76) |  | 0.88 (0.70, 1.10) | 0.26 |  | 0.94 (0.74, 1.20) | 0.63 |  | 156 (95) | 295 (95) | 0.47 (0.16, 1.41) | 0.18 |
| *Faecalitalea* | 0.04 (0.00, 6.39) | 0.000 (0.00, 6.41) |  | 1.17 (0.94, 1.45) | 0.17 |  | 1.07 (0.86, 1.32) | 0.56 |  | 98 (59) | 155 (50) | 1.76 (1.09, 2.84) | 0.02 |
| *Flavonifractor* | 0.10 (0.00, 1.43) | 0.092 (0.00, 1.27) |  | 1.00 (0.80, 1.27) | 0.97 |  | 1.13 (0.91, 1.41) | 0.27 |  | 138 (84) | 247 (79) | 1.27 (0.71, 2.26) | 0.42 |
| *Fusicatenibacter* | 0.21 (0.00, 10.23) | 0.273 (0.00, 10.19) |  | 0.99 (0.78, 1.25) | 0.92 |  | 0.99 (0.80, 1.23) | 0.94 |  | 137 (83) | 250 (80) | 1.00 (0.56, 1.78) | 1.00 |
| *GCA-900066575* | 0.04 (0.00, 0.80) | 0.036 (0.00, 1.91) |  | 1.02 (0.82, 1.28) | 0.83 |  | 1.09 (0.84, 1.41) | 0.53 |  | 112 (68) | 193 (62) | 1.23 (0.77, 1.98) | 0.39 |
| *Haemophilus* | 0.12 (0.00, 38.24) | 0.131 (0.00, 45.70) |  | 0.97 (0.78, 1.20) | 0.77 |  | 1.03 (0.83, 1.27) | 0.78 |  | 134 (81) | 249 (80) | 1.15 (0.65, 2.05) | 0.63 |
| *Hungatella* | 0.02 (0.00, 1.04) | 0.012 (0.00, 2.45) |  | 0.92 (0.73, 1.16) | 0.48 |  | 1.01 (0.78, 1.30) | 0.96 |  | 96 (58) | 159 (51) | 1.42 (0.91, 2.20) | 0.12 |
| *Intestinibacter* | 0.08 (0.00, 22.95) | 0.079 (0.00, 43.82) |  | 1.02 (0.82, 1.28) | 0.83 |  | 1.11 (0.83, 1.48) | 0.49 |  | 116 (70) | 209 (67) | 1.17 (0.71, 1.92) | 0.53 |
| *Lachnoclostridium* | 1.38 (0.03, 17.70) | 1.390 (0.00, 18.06) |  | 1.00 (0.80, 1.27) | 0.98 |  | 1.05 (0.83, 1.32) | 0.69 |  | 165 (100) | 308 (99) | - | - |
| *Lachnospira* | 0.04 (0.00, 1.79) | 0.027 (0.00, 4.05) |  | 1.09 (0.86, 1.38) | 0.48 |  | 0.98 (0.72, 1.33) | 0.88 |  | 98 (59) | 185 (59) | 1.02 (0.63, 1.65) | 0.94 |
| *Lachnospiraceae FCS020 group* | 0.05 (0.00, 1.74) | 0.029 (0.00, 1.32) |  | 1.03 (0.82, 1.29) | 0.82 |  | 1.13 (0.91, 1.41) | 0.28 |  | 107 (65) | 182 (59) | 1.34 (0.82, 2.20) | 0.24 |
| *Lachnospiraceae NK4A136 group* | 0.34 (0.00, 3.46) | 0.285 (0.00, 5.73) |  | 0.89 (0.72, 1.11) | 0.30 |  | 0.89 (0.69, 1.15) | 0.38 |  | 149 (90) | 254 (82) | 2.27 (1.10, 4.69) | 0.03 |
| *Lachnospiraceae UCG-004* | 0.31 (0.00, 3.74) | 0.281 (0.00, 4.16) |  | 0.93 (0.73, 1.17) | 0.53 |  | 0.95 (0.75, 1.20) | 0.66 |  | 134 (81) | 241 (77) | 0.92 (0.51, 1.67) | 0.79 |
| *Lachnospiraceae UCG-010* | 0.03 (0.00, 5.87) | 0.010 (0.00, 5.35) |  | 1.12 (0.91, 1.39) | 0.28 |  | 1.13 (0.92, 1.38) | 0.24 |  | 93 (56) | 158 (51) | 1.25 (0.79, 1.96) | 0.34 |
| *Negativibacillus* | 0.04 (0.00, 2.87) | 0.029 (0.00, 3.52) |  | 1.01 (0.79, 1.28) | 0.96 |  | 0.98 (0.78, 1.23) | 0.84 |  | 98 (59) | 186 (60) | 0.99 (0.63, 1.55) | 0.95 |
| *Odoribacter* | 0.04 (0.00, 4.54) | 0.034 (0.00, 2.70) |  | 1.02 (0.82, 1.27) | 0.84 |  | 0.98 (0.81, 1.20) | 0.86 |  | 102 (62) | 186 (60) | 0.86 (0.55, 1.36) | 0.53 |
| *Parabacteroides* | 0.43 (0.00, 14.18) | 0.336 (0.00, 12.68) |  | 1.04 (0.82, 1.30) | 0.76 |  | 1.09 (0.90, 1.33) | 0.36 |  | 124 (75) | 241 (77) | 0.70 (0.42, 1.15) | 0.16 |
| *Parasutterella* | 0.01 (0.00, 6.53) | 0.012 (0.00, 4.15) |  | 1.00 (0.80, 1.24) | 0.98 |  | 0.98 (0.81, 1.20) | 0.88 |  | 85 (52) | 164 (53) | 0.86 (0.55, 1.34) | 0.49 |
| *Phascolarctobacterium* | 0.09 (0.00, 1.16) | 0.101 (0.00, 1.54) |  | 0.78 (0.64, 0.95) | 0.02 |  | 0.89 (0.72, 1.10) | 0.29 |  | 112 (68) | 195 (63) | 0.94 (0.59, 1.50) | 0.78 |
| *Roseburia* | 1.27 (0.00, 18.67) | 1.020 (0.00, 18.16) |  | 1.09 (0.85, 1.40) | 0.48 |  | 0.92 (0.72, 1.16) | 0.46 |  | 150 (91) | 278 (89) | 1.47 (0.68, 3.20) | 0.33 |
| *Ruminiclostridium 5* | 0.44 (0.00, 2.78) | 0.439 (0.00, 2.55) |  | 1.06 (0.83, 1.35) | 0.66 |  | 1.00 (0.79, 1.25) | 0.97 |  | 158 (96) | 308 (99) | - | - |
| *Ruminiclostridium 9* | 0.20 (0.00, 2.24) | 0.182 (0.00, 4.75) |  | 1.05 (0.82, 1.34) | 0.69 |  | 1.03 (0.78, 1.36) | 0.82 |  | 146 (88) | 273 (88) | 1.11 (0.54, 2.29) | 0.78 |
| *Ruminococcaceae UCG-002* | 0.15 (0.00, 2.93) | 0.153 (0.00, 4.54) |  | 0.98 (0.80, 1.19) | 0.82 |  | 0.96 (0.77, 1.19) | 0.73 |  | 121 (73) | 208 (67) | 1.28 (0.77, 2.13) | 0.34 |
| *Ruminococcaceae UCG-003* | 0.03 (0.00, 1.09) | 0.022 (0.00, 1.63) |  | 1.04 (0.84, 1.29) | 0.72 |  | 1.02 (0.80, 1.29) | 0.88 |  | 97 (59) | 169 (54) | 1.08 (0.68, 1.71) | 0.75 |
| *Ruminococcaceae UCG-004* | 0.07 (0.00, 0.77) | 0.068 (0.00, 0.77) |  | 0.97 (0.77, 1.22) | 0.80 |  | 0.97 (0.78, 1.21) | 0.78 |  | 103 (62) | 192 (62) | 0.99 (0.62, 1.58) | 0.96 |
| *Ruminococcaceae UCG-005* | 0.03 (0.00, 2.36) | 0.023 (0.00, 8.10) |  | 1.03 (0.83, 1.27) | 0.79 |  | 0.84 (0.63, 1.12) | 0.24 |  | 101 (61) | 181 (58) | 1.01 (0.64, 1.60) | 0.98 |
| *Streptococcus* | 0.34 (0.00, 24.44) | 0.325 (0.00, 41.95) |  | 0.93 (0.74, 1.18) | 0.56 |  | 0.90 (0.67, 1.21) | 0.49 |  | 158 (96) | 308 (99) | - | - |
| *Subdoligranulum* | 1.49 (0.00, 18.36) | 1.211 (0.00, 20.15) |  | 1.11 (0.89, 1.40) | 0.35 |  | 1.10 (0.88, 1.37) | 0.42 |  | 144 (87) | 254 (82) | 1.29 (0.68, 2.45) | 0.44 |
| *Sutterella* | 0.01 (0.00, 12.70) | 0.061 (0.00, 9.96) |  | 0.98 (0.78, 1.23) | 0.83 |  | 1.04 (0.86, 1.26) | 0.67 |  | 83 (50) | 175 (56) | 0.75 (0.48, 1.16) | 0.20 |
| *Tyzzerella* | 0.00 (0.00, 10.69) | 0.008 (0.00, 11.59) |  | 1.09 (0.86, 1.38) | 0.46 |  | 1.00 (0.79, 1.28) | 0.97 |  | 82 (50) | 157 (50) | 0.86 (0.55, 1.35) | 0.51 |
| *UBA1819* | 0.05 (0.00, 1.80) | 0.042 (0.00, 3.60) |  | 0.97 (0.78, 1.21) | 0.80 |  | 1.03 (0.81, 1.30) | 0.82 |  | 122 (74) | 217 (70) | 1.30 (0.78, 2.17) | 0.31 |
| *uncultured* | 0.51 (0.00, 2.87) | 0.454 (0.00, 6.16) |  | 1.20 (0.95, 1.52) | 0.13 |  | 1.03 (0.81, 1.32) | 0.79 |  | 157 (96) | 285 (92) | - | - |
| *Veillonella* | 0.03 (0.00, 2.55) | 0.041 (0.00, 4.24) |  | 0.98 (0.77, 1.23) | 0.84 |  | 0.82 (0.61, 1.11) | 0.20 |  | 110 (67) | 229 (74) | 0.74 (0.45, 1.20) | 0.22 |

Abbreviations: OR: Odds ratios; 95%CI: 95% Confidence Intervals; *A Priori*: Bacteria determined the literature to be related to colorectal cancer incidence.

^a^ Conditional logistic regression models adjusted for age, year of colonoscopy, regular non-steroidal anti-inflammatory drug and/or aspirin use (yes/no), current alcohol use (yes/no), family history of polyps (yes/no), education (high school education or less; or some college or more), hormone replacement therapy use (yes/no), daily fiber intake (g/day), body mass index (kg/m^2^), total daily energy intake (kcal/day), physical activity level (no moderate or vigorous activity, unknown, missing; moderate activity, or vigorous activity), race (Black, White, other), smoking status (current/former smokers or never smokers), red and processed meat intake (g/day), and study center (Walter Reed, San Diego, Portsmouth, or Bethesda).

^b^ Bacterial relative abundances were transformed using the center log ratio transformation and standardized by *X*-mean of the control group divided by the standard deviation of the control group; *A priori* bacteria that were not present in ≥50% of samples at an average relative abundance of ≥0.01% are not included

^c^ Bacterial relative abundances were transformed by multiplying by 100 and standardized by *X*-mean of the control group divided by the standard deviation of the control group; *A priori* bacteria that were not present in ≥50% of samples at an average relative abundance of ≥0.01% are not included

^d^ Bacteria prevalent in 5%-95% of total sample size (n=476).

**Supplemental Table 8**. Associations^a^ of co-abundance groups with adenomas among N=165 adenoma cases and N=311 controls in the Colorectal Neoplasia Screening with Colonoscopy in Average-Risk Women Regional Navy/Army Medical Centers study, 2000-2002

| Co-abundance group^b^ | Comprising Phylum | OR (95% CI) | *P* |
| --- | --- | --- | --- |
| G1 | Firmicutes and Actinobacteria | 1.01 (0.99, 1.02) | 0.33 |
| G2 | Firmicutes and Verrucomicrobia | 0.98 (0.87, 1.10) | 0.77 |
| G3 | Firmicutes, Fusobacteria, Proteobacteria, Epsilonbacteraeota, and Actinobacteria | 0.96 (0.91, 1.01) | 0.15 |
| G4 | Firmicutes, Verrucomicrobia, Proteobacteria, Bacteroidetes, Cyanobacteria, and Actinobacteria | 0.97 (0.92, 1.02) | 0.18 |
| G5 | Firmicutes and Bacteroidetes | 0.97 (0.84, 1.12) | 0.67 |
| G6 | Firmicutes, Proteobacteria, Verrucomicrobia, Bacteroidetes, and Actinobacteria | 1.00 (0.98, 1.03) | 0.71 |
| G7 | Firmicutes and Actinobacteria | 1.00 (0.97, 1.03) | 0.94 |
| G8 | Firmicutes, Proteobacteria, Bacteroidetes, and Actinobacteria | 1.00 (0.98, 1.02) | 0.87 |
| G9 | Firmicutes | 0.97 (0.92, 1.02) | 0.24 |
| G10 | Firmicutes, Proteobacteria, Fusobacteria | 0.99 (0.93, 1.07) | 0.85 |
| G11 | Firmicutes | 0.89 (0.76, 1.04) | 0.14 |
| G12 | Firmicutes | 1.03 (0.95, 1.11) | 0.47 |
| G13 | Firmicutes | 1.00 (0.99, 1.02) | 0.68 |
| G14 | Firmicutes, Bacteroidetes, and Proteobacteria | 1.02 (0.97, 1.07) | 0.47 |
| G15 | Firmicutes | 0.96 (0.89, 1.03) | 0.28 |
| G16 | Firmicutes and Bacteroidetes | 0.96 (0.86, 1.07) | 0.42 |
| G17 | Firmicutes and Proteobacteria | 1.00 (0.96, 1.04) | 0.99 |

^a^ Conditional logistic regression models adjusted for age, year of colonoscopy, regular non-steroidal anti-inflammatory drug and/or aspirin use (yes/no), current alcohol use (yes/no), family history of polyps (yes/no), education (high school education or less; or some college or more), hormone replacement therapy use (yes/no), daily fiber intake (g/day), body mass index (kg/m^2^), total daily energy intake (kcal/day), physical activity level (no moderate or vigorous activity, unknown, missing; moderate activity, or vigorous activity), race (Black, White, other), smoking status (current/former smokers or never smokers), red and processed meat intake (g/day), and study center (Walter Reed, San Diego, Portsmouth, or Bethesda).

^b^ Characterized by the sum of counts over all amplicon sequence variants within the co-abundance group. A total of 363 bacterial ASVs present in at least 5% of the samples were clustered into co-abundance groups (CAGs) by using a hierarchical cluster analysis based on SparCC co-abundance correlations as a taxon-wise distance metric. Co-abundance groups were determined by examining the gap statistic, which resulted in 17 guilds.

Abbreviations: G, Co-abundance guild

**Supplemental Table 9.** Associations of circulating bile acids with adenomas among N=164 adenoma cases and N=328 controls in the Colorectal Neoplasia Screening with Colonoscopy in Average-Risk Women Regional Navy/Army Medical Centers study, 2000-2002

|  |  |  |  |  | Adenoma Characteristic a | | | |
| --- | --- | --- | --- | --- | --- | --- | --- | --- |
|  |  | Range | No. cases/control | Overall | Advanced Adenoma | Normal Adenoma | Distal | Proximal |
| **Summary Score Bile Acids** |  |  |  |  |  |  |  |  |
| Total | |  |  |  |  |  |  |  |
|  | T1 | (3.87, 115.42) | 51/108 | 1.00 | 1.00 | 1.00 | 1.00 | 1.00 |
|  | T2 | (115.72, 285.72) | 47/108 | 0.87 (0.50, 1.51) | 1.05 (0.52, 2.13) | 0.92 (0.51, 1.68) | 1.10 (0.55, 2.21) | 0.87 (0.47, 1.62) |
|  | T3 | (289.14, 12598.63 | 66/112 | 1.39 (0.82, 2.36) | 1.40 (0.71, 2.76) | 1.55 (0.88, 2.76) | 1.41 (0.71, 2.79) | 1.51 (0.83, 2.74) |
|  | *P-trend* |  |  | 0.1 | 0.31 | 0.08 | 0.30 | 0.09 |
|  | *P-heterogeneity* |  |  |  | 0.42 | *Ref.* | 0.92 | *Ref.* |
| Primaryb | |  |  |  |  |  |  |  |
|  | T1 | (3.76, 71.81) | 48/108 | 1.00 | 1.00 | 1.00 | 1.00 | 1.00 |
|  | T2 | (72.72, 198.95) | 50/108 | 0.96 (0.55, 1.67) | 1.26 (0.63, 2.55) | 1.00 (0.55, 1.82) | 1.25 (0.63, 2.50) | 0.99 (0.54, 1.82) |
|  | T3 | (199.09, 11573.60 | 66/112 | 1.45 (0.86, 2.45) | 1.53 (0.77, 3.04) | 1.61 (0.91, 2.85) | 1.66 (0.85, 3.23) | 1.49 (0.83, 2.69) |
|  | *P-trend* |  |  | 0.10 | 0.26 | 0.07 | 0.15 | 0.14 |
|  | *P-heterogeneity* |  |  |  | 0.40 | *Ref.* | 0.95 |  |
| Secondaryb | |  |  |  |  |  |  |  |
|  | T1 | (0.09, 115.59) | 48/108 | 1.00 | 1.00 | 1.00 | 1.00 | 1.00 |
|  | T2 | (12.54, 94.81) | 56/108 | 1.16 (0.69, 1.97) | 1.03 (0.51, 2.08) | 1.33 (0.74, 2.41) | 1.55 (0.79, 3.05) | 0.99 (0.54, 1.82) |
|  | T3 | (37.13, 1038.44) | 60/112 | 1.15 (0.66, 2.01) | 1.16 (0.58, 2.33) | 1.31 (0.72, 2.37) | 1.30 (0.64, 2.62) | 1.18 (0.65, 2.15) |
|  | *P-trend* |  |  | 0.72 | 0.66 | 0.51 | 0.70 | 0.53 |
|  | *P-heterogeneity* |  |  |  | 0.70 | *Ref.* | 0.79 | *Ref.* |
| **Individual Bile Acids** | |  |  |  |  |  |  |  |
| Cholate | |  |  |  |  |  |  |  |
|  | T1 | (0.01, 2.14) | 63/108 | 1.00 | 1.00 | 1.00 | 1.00 | 1.00 |
|  | T2 | (2.15, 5.64) | 44/108 | 0.77 (0.45, 1.30) | 0.57 (0.28, 1.17) | 0.85 (0.48, 1.51) | 0.91 (0.47, 1.78) | 0.61 (0.33, 1.14) |
|  | T3 | (5.68, 390.00) | 57/112 | 0.92 (0.56, 1.52) | 0.91 (0.47, 1.75) | 0.99 (0.56, 1.75) | 0.93 (0.47, 1.81) | 0.95 (0.53, 1.70) |
|  | *P-trend* |  |  | 0.62 | 0.59 | 0.91 | 0.81 | 0.69 |
|  | *P-heterogeneity* |  |  |  | 0.54 | *Ref.* | 0.04 | *Ref.* |
| Deoxycholate | |  |  |  |  |  |  |  |
|  | T1 | (0.01, 1.68) | 48/108 | 1.00 | 1.00 | 1.00 | 1.00 | 1.00 |
|  | T2 | (1.69, 12.90) | 51/108 | 1.03 (0.60, 1.76) | 1.28 (0.63, 2.62) | 1.06 (0.58, 1.93) | 1.08 (0.54, 2.14) | 1.18 (0.64, 2.19) |
|  | T3 | (13.20, 520.00) | 65/112 | 1.21 (0.70, 2.09) | 1.30 (0.63, 2.66) | 1.34 (0.75, 2.41) | 1.23 (0.62, 2.43) | 1.38 (0.75, 2.55) |
|  | *P-trend* |  |  | 0.59 | 0.43 | 0.41 | 0.60 | 0.33 |
|  | *P-heterogeneity* |  |  |  | 0.79 | *Ref.* | 0.54 | *Ref.* |
| Chenodeoxycholate | |  |  |  |  |  |  |  |
|  | T1 | (0.01, 1.41) | 52/108 | 1.00 | 1.00 | 1.00 | 1.00 | 1.00 |
|  | T2 | (1.42, 5.56) | 49/108 | 0.85 (0.49, 1.48) | 1.10 (0.55, 2.20) | 0.89 (0.49, 1.60) | 0.93 (0.47, 1.82) | 0.99 (0.54, 1.80) |
|  | T3 | (5.71, 1270.00) | 63/112 | 1.14 (0.67, 1.93) | 1.17 (0.58, 2.36) | 1.16 (0.66, 2.05) | 1.12 (0.57, 2.17) | 1.16 (0.64, 2.10) |
|  | *P-trend* |  |  | 0.72 | 0.66 | 0.68 | 0.80 | 0.65 |
|  | *P-heterogeneity* |  |  |  | 0.71 | *Ref.* | 0.71 | *Ref.* |
| Glycochenodeoxycholate | |  |  |  |  |  |  |  |
|  | T1 | (0.81, 39.30) | 50/107 | 1.00 | 1.00 | 1.00 | 1.00 | 1.00 |
|  | T2 | (39.50, 120.00) | 55/109 | 1.19 (0.69, 2.07) | 1.78 (0.88, 3.60) | 0.85 (0.47, 1.53) | 1.40 (0.71, 2.75) | 0.97 (0.54, 1.75) |
|  | T3 | (121.00, 3520.00) | 59/112 | 1.14 (0.68, 1.93) | 1.40 (0.67, 2.93) | 1.15 (0.65, 2.02) | 1.47 (0.76, 2.88) | 1.06 (0.59, 1.91) |
|  | *P-trend* |  |  | 0.62 | 0.34 | 0.66 | 0.25 | 0.86 |
|  | *P-heterogeneity* |  |  |  | 0.54 | *Ref.* | 0.76 | *Ref.* |
| Glycocholate | |  |  |  |  |  |  |  |
|  | T1 | (0.35, 10.80) | 54/107 | 1.00 | 1.00 | 1.00 | 1.00 | 1.00 |
|  | T2 | (10.90, 28.40) | 52/109 | 1.06 (0.63, 1.79) | 1.00 (0.51, 1.99) | 1.02 (0.57, 1.82) | 1.22 (0.63, 2.35) | 0.87 (0.48, 1.60) |
|  | T3 | (29.10, 6020.00) | 58/112 | 1.33 (0.79, 2.26) | 1.23 (0.63, 2.41) | 1.21 (0.68, 2.14) | 1.17 (0.61, 2.25) | 1.23 (0.69, 2.21) |
|  | *P-trend* |  |  | 0.28 | 0.55 | 0.51 | 0.64 | 0.48 |
|  | *P-heterogeneity* |  |  |  | 0.38 | *Ref.* | 0.97 | *Ref.* |
| Glycodeoxycholate | |  |  |  |  |  |  |  |
|  | T1 | (0.06, 12.60) | 45/108 | 1.00 | 1.00 | 1.00 | 1.00 | 1.00 |
|  | T2 | (12.70, 34.10) | 54/108 | 1.02 (0.59, 1.76) | 1.15 (0.57, 2.36) | 1.17 (0.64, 2.13) | 1.54 (0.76, 3.13) | 0.93 (0.50, 1.72) |
|  | T3 | (34.60, 769.00) | 65/112 | 1.28 (0.75, 2.18) | 1.41 (0.70, 2.83) | 1.53 (0.86, 2.74) | 1.67 (0.83, 3.36) | 1.35 (0.75, 2.42) |
|  | *P-trend* |  |  | 0.34 | 0.33 | 0.14 | 0.15 | 0.29 |
|  | *P-heterogeneity* |  |  |  | 0.67 | *Ref.* | 0.65 | *Ref.* |
| Glycolithocholatec | |  |  |  |  |  |  |  |
|  | T1 | (0.00, 0.01) | 108/216 | 1.00 | 1.00 | 1.00 | 1.00 | 1.00 |
|  | T2 | (0.01, 32.20) | 56/112 | 1.43 (1.24, 1.64) | 1.07 (0.59, 1.92) | 0.81 (0.49, 1.34) | 0.82 (0.45, 1.48) | 0.93 (0.56, 1.56) |
|  | *P-trend* |  |  | 0.44 | 0.82 | 0.39 | 0.5 | 0.78 |
|  | *P-heterogeneity* |  |  |  | 0.18 | *Ref.* | 0.76 | *Ref.* |
| Glycoursodeoxycholate | |  |  |  |  |  |  |  |
|  | T1 | (0.01, 4.37) | 62/108 | 1.00 | 1.00 | 1.00 | 1.00 | 1.00 |
|  | T2 | (4.38, 13.50) | 39/108 | 0.62 (0.36, 1.08) | 0.75 (0.37, 1.51) | 0.51 (0.28, 0.94) | 0.81 (0.41, 1.62) | 0.48 (0.26, 0.90) |
|  | T3 | (13.90, 359.00) | 63/112 | 0.91 (0.56, 1.50) | 1.01 (0.51, 1.98) | 0.99 (0.57, 1.70) | 1.18 (0.61, 2.27) | 0.89 (0.51, 1.56) |
|  | *P-trend* |  |  | 0.71 | 0.99 | 0.94 | 0.62 | 0.65 |
|  | *P-heterogeneity* |  |  |  | 0.95 | *Ref.* | 0.62 | *Ref.* |
| Lithocholatec | |  |  |  |  |  |  |  |
|  | T1 | (0.01, 0.01) | 127/262 | 1.00 | 1.00 | 1.00 | 1.00 | 1.00 |
|  | T2 | (0.02, 8.16) | 37/66 | 1.07 (0.61, 1.87) | 1.21 (0.61, 2.38) | 1.12 (0.63, 2.00) | 1.78 (0.92, 3.41) | 0.80 (0.42, 1.51) |
|  | *P-trend* |  |  | 0.81 | 0.57 | 0.69 | 0.07 | 0.45 |
|  | *P-heterogeneity* |  |  |  | 0.81 | *Ref.* | 0.01 | *Ref.* |
| Taurochenodeoxycholate | |  |  |  |  |  |  |  |
|  | T1 | (0.02, 5.93) | 44/108 | 1.00 | 1.00 | 1.00 | 1.00 | 1.00 |
|  | T2 | (5.98, 19.50) | 55/108 | 1.43 (0.82, 2.50) | 1.33 (0.67, 2.64) | 1.46 (0.79, 2.69) | 1.03 (0.51, 2.09) | 1.68 (0.92, 3.09) |
|  | T3 | (19.60, 765.00) | 65/112 | 1.55 (0.93, 2.60) | 1.19 (0.60, 2.39) | 2.03 (1.12, 3.67) | 1.75 (0.92, 3.32) | 1.59 (0.86, 2.96) |
|  | *P-trend* |  |  | 0.10 | 0.64 | 0.02 | 0.08 | 0.17 |
|  | *P-heterogeneity* |  |  |  | 0.12 | *Ref.* | 0.84 | *Ref.* |
| Taurocholate | |  |  |  |  |  |  |  |
|  | T1 | (0.01, 1.12) | 48/107 | 1.00 | 1.00 | 1.00 | 1.00 | 1.00 |
|  | T2 | (1.13, 5.26) | 54/109 | 1.34 (0.76, 2.34) | 1.34 (0.68, 2.64) | 1.15 (0.62, 2.13) | 1.08 (0.54, 2.17) | 1.30 (0.71, 2.40) |
|  | T3 | (5.29, 2570.00) | 62/112 | 1.59 (0.92, 2.77) | 1.07 (0.53, 2.17) | 1.76 (0.98, 3.18) | 1.54 (0.80, 2.94) | 1.42 (0.77, 2.64) |
|  | *P-trend* |  |  | 0.10 | 0.81 | 0.06 | 0.21 | 0.27 |
|  | *P-heterogeneity* |  |  |  | 0.06 | *Ref.* | 0.80 | *Ref.* |
| Taurodeoxycholate | |  |  |  |  |  |  |  |
|  | T1 | (0.00, 1.13) | 47/108 | 1.00 | 1.00 | 1.00 | 1.00 | 1.00 |
|  | T2 | (1.16, 5.77) | 50/108 | 1.18 (0.68, 2.02) | 0.78 (0.39, 1.58) | 1.45 (0.79, 2.66) | 0.93 (0.44, 1.95) | 1.27 (0.70, 2.31) |
|  | T3 | (5.79, 334.00) | 67/112 | 1.52 (0.89, 2.58) | 1.17 (0.60, 2.27) | 1.91 (1.05, 3.48) | 2.05 (1.07, 3.95) | 1.26 (0.68, 2.31) |
|  | *P-trend* |  |  | 0.17 | 0.95 | 0.04 | 0.12 | 0.39 |
|  | *P-heterogeneity* |  |  |  | 0.21 | *Ref.* | 0.22 | *Ref.* |
| Ursodeoxycholate | |  |  |  |  |  |  |  |
|  | T1 | (0.01, 21.10) | 63/108 | 1.00 | 1.00 | 1.00 | 1.00 | 1.00 |
|  | T2 | (0.01, 51.10) | 44/108 | 0.77 (0.45, 1.30) | 0.57 (0.28, 1.17) | 0.85 (0.48, 1.51) | 0.91 (0.47, 1.78) | 0.61 (0.33, 1.14) |
|  | T3 | (0.01, 163.00) | 57/112 | 0.92 (0.56, 1.52) | 0.91 (0.47, 1.75) | 0.99 (0.56, 1.75) | 0.93 (0.47, 1.81) | 0.95 (0.53, 1.70) |
|  | *P-trend* |  |  | 0.62 | 0.59 | 0.91 | 0.81 | 0.69 |
|  | *P-heterogeneity* |  |  |  | 0.86 | *Ref.* | 0.86 | *Ref.* |

^a^ Conditional logistic regression models adjusted for age, year of colonoscopy, regular non-steroidal anti-inflammatory drug and/or aspirin use (yes/no), current alcohol use (yes/no), family history of polyps (yes/no), education (high school education or less; or some college or more), hormone replacement therapy use (yes/no), daily fiber intake (g/day), body mass index (kg/m^2^), total daily energy intake (kcal/day), physical activity level (no moderate or vigorous activity, unknown, missing; moderate activity, or vigorous activity), race (Black, White, other), smoking status (current/former smokers or never smokers), red and processed meat intake (g/day), and study center (Walter Reed, San Diego, Portsmouth, or Bethesda).

^b^ Primary Bile Acids = log2 of the sum chenodeoxycholic acid, cholic acid, glycocholic acid, glycochenodeoxycholic acid, taurocholic acid, and taurochenodeoxycholic acid; secondary Bile Acids =log2 of the sum of deoxycholic acid, lithocholic acid, glycodeoxycholic acid, glycolithocholic acid, and taurodeoxycholic acid

^c^ These bile acids were dichotomized due to a higher number of metabolites below the limit of detection
